# Complete reference genome assemblies and annotations of three *Escherichia coli* MRE162 clones

**DOI:** 10.1128/MRA.00490-23

**Published:** 2023-10-09

**Authors:** Russell J. S. Orr, Eloi Littner, Guilhem Larigauderie, Claire L. Lonsdale, Marius Dybwad

**Affiliations:** 1 Total Defence, Norwegian Defence Research Establishment (FFI), Kjeller, Norway; 2 Division Biologie, DGA Maîtrise NRBC, Vert-le-Petit, France; 3 CBR Division, Dstl, Porton Down, Salisbury, United Kingdom; University of Maryland School of Medicine, Baltimore, Maryland, USA

**Keywords:** *Escherichia coli*, DNA sequencing, genome analysis

## Abstract

*Escherichia coli* MRE162 was originally isolated from a toilet pan in 1949 and since been utilized in numerous studies. Here, we sequence, assemble, and annotate clones held at three laboratories providing reference-level assemblies. We show the uniqueness of MRE162 to strains in open databases and make the UK clone publically available.

## ANNOUNCEMENT

*Escherichia coli* MRE162 was isolated from a toilet pan at Porton Down, Salisbury, UK, in 1949, freeze-dried in a glass ampoule in 1967, and stored at −20°C. The strain has been characterized in depth regarding aerosol survival and “Open Air Factor” interaction (1 – 6). The original clone was gifted to DGA, France, in 2002 and FFI, Norway, in 2014 for separate studies at these laboratories. We now retrospectively check for mutations between the three clones, which could potentially impact comparisons between studies.

**Fig 1:**
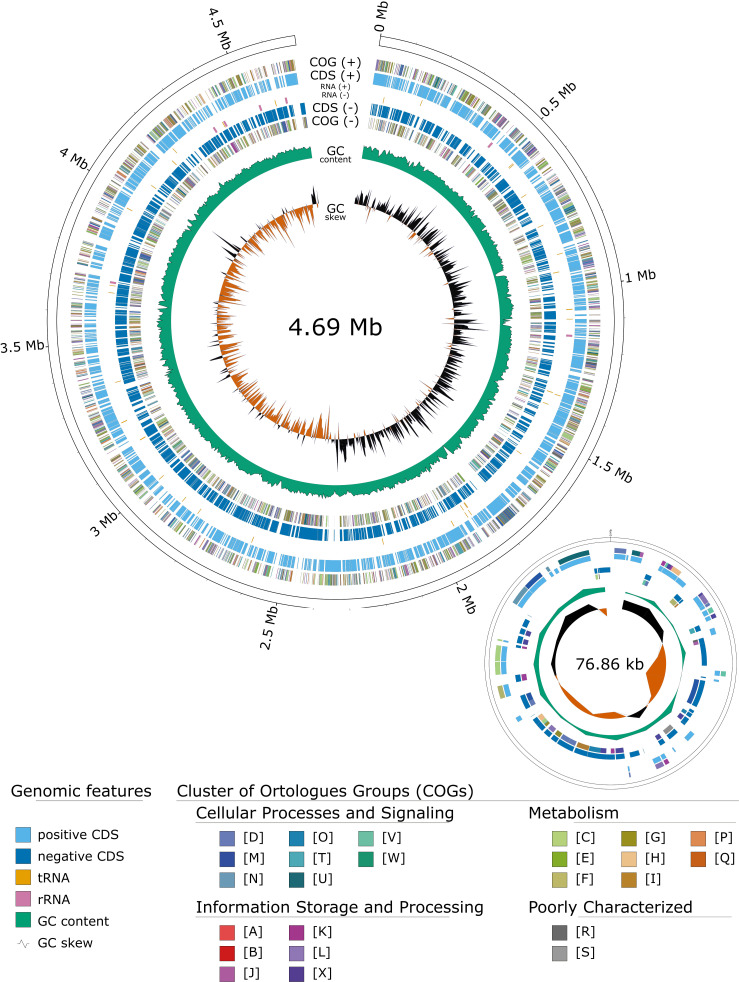
Genome map of MRE162 UK clone. Circular representation of the MRE162 UK clone genome. The upper map shows the chromosome, with the lower depicting the plasmid. Genomic features and Cluster of Orthologs Groups (COGs) with corresponding keys are mapped on the genome representation. Genomic features and COGs obtained from PGAP annotation.

MRE162 clones were cultured separately at each laboratory overnight at 37°C on LB broth, TSA, or rich media agar plates. DNA was isolated using the Qiagen DNeasy Blood and Tissue Kit (FRA, NOR) or lysozyme and RNase lysis followed by proteinase K treatment and SPRI bead purification (UK). Eluted gDNA was quantified on a Qubit Fluorometer (Invitrogen). DNA was sheared to 350 bp (NOR), and libraries were constructed with Illumina DNA Prep Kit (NOR) or Nextera XT Prep Kit (average insert size 550 bp: FRA, UK). Libraries were sequenced on Miniseq (NOR) or MiSeq (FRA, UK). QC was performed with FastQC v0.12.0 (7), and reads were trimmed using TrimGalore v0.6.10 (8) with –length 80 and –q 30. For Nanopore sequencing, libraries were constructed from the same unsheared DNA without size selection using the Ligation Sequencing Kit (UK), Rapid Sequencing Kit (FRA), or Rapid Barcoding Kit (NOR) and sequenced on R9.4.1 flow cells. Basecalling was performed with high accuracy (UK) or super accuracy (FRA, NOR) with Guppy v15.0.0 (9). QC was performed with nanoQC v2.8.0 (10), and reads were trimmed using NanoFilt v2.8.0 (10) with -q 10 L 1000. Trimmed Nanopore reads were error corrected using FMLRC2 v0.1.7 (11) with cache size 10 and k-mer sizes 21,59,79,127. Hybrid *de novo* assemblies of MRE162 clones were performed using Unicycler v0.5.0 (12) in “conservative” mode, allowing circularization of overlapping ends, confirmed with assembly graphs, and rotating assemblies to begin at a consistent starting gene (dnaA). Assemblies were polished with Polypolish v0.5.0 (13) and annotated with PGAP v2022-12-13.build6494 (14). A genome map (Fig 1) of MRE162-UK was constructed in GenoVi v0.2.16 (15). Chromosome and plasmid sequences were compared, including the closest blastn NCBInt hits (as of January 2023), with nucmer v4.0.0.rc1 (16). Assembly statistics were obtained using Bowtie2 v2.5.1 (17) and Minimap2 v2.24-r1122 (18). Default parameters were used for all software unless otherwise specified.

The reads were assembled to complete circularized chromosomes and plasmids. Chromosome and plasmid length were comparable (Fig. 1 and Table 1) between the three clones, except for A 5.1 kb deletion noted in the FRA clone chromosome. Comparing chromosomes and plasmids at the nucleotide level shows >99.9% identity between clones compared to 96.6% identity to the closest identified *E. coli* chromosome (strain S30: CP010231) and 82.39% identity to the closest plasmid (strain S30: CP010234). Highlighting the uniqueness of the MRE162 plasmid, only 45.18% of its length aligned with the S30 plasmid. These genome assemblies and annotations, alongside the publicly available culture, provide invaluable data for future evolutionary and functional studies.

**TABLE 1:** Assembly statistics, sequence data and genome annotation^*a*^

Assembly	MRE162 UK chromosome	MRE162 UK plasmid	MRE162 FRA chromosome	MRE162 FRA plasmid	MRE162 NOR chromosome	MRE162 NOR plasmid
GenBank accession	CP119404	CP119405	CP119402	CP119403	CP119400	CP119401
Size (bp)	4,690,036	76,855	4,684,699	76,849	4,690,371	76,850
GC %	50.6	47.9	50.6	47.9	50.6	47.9
Illumina coverage	91x	126x	39x	37x	121x	117x
ONT coverage	46x	26x	734x	902x	194x	316x
Sequence data	MRE162 UK genome		MRE162 FRA genome		MRE162 NOR genome	
BioSample accession	SAMN33417455		SAMN33417456		SAMN33417457	
SRA accession Illumina	SRX20287199		SRX20287201		SRX20287203	
Illumina reads (PE)	963,103		857,846		2,396,231	
SRA accession ONT	SRX20287200		SRX20287202		SRX20287204	
ONT reads	29,076		931,215		153,568	
ONT N50	26,718		8,265		11,777	
Annotation	MRE162 UK genome		MRE162 FRA genome		MRE162 NOR genome	
Genes (total)	4,645		4,638		4,640	
CDSs (total)	4,524		4,517		4,519	
Genes (coding)	4,320		4,312		4,314	
CDSs (with protein)	4,320		4,312		4,314	
Genes (RNA)	121		121		121	
rRNAs	8, 7, 7 (5S, 16S, 23S)		8, 7, 7 (5S, 16S, 23S)		8, 7, 7 (5S, 16S, 23S)	
Complete rRNAs	8, 7, 7 (5S, 16S, 23S)		8, 7, 7 (5S, 16S, 23S)		8, 7, 7 (5S, 16S, 23S)	
tRNAs	86		86		86	
ncRNAs	13		13		13	
Pseudo genes (total)	204		205		205	
CDSs (without protein)	204		205		205	
Pseudo genes (ambiguous residues)	0 of 204		0 of 205		0 of 205	
Pseudo genes (frameshifted)	56 of 204		56 of 205		54 of 205	
Pseudo genes (incomplete)	133 of 204		134 of 205		136 of 205	
Pseudo genes (internal stop)	58 of 204		58 of 205		58 of 205	
Pseudo genes (multiple problems)	35 of 204		35 of 205		35 of 205	

^*a*^ Statistics from Bowtie2 and Minimap2 for both the chromosome and plasmid of the three MRE162 clones. Annotation for each complete genome from PGAP.

## Data Availability

The complete assemblies and annotations of the three *E. coli* MRE162 clones (FRA, NOR, and UK) have been deposited in GenBank under the following accession numbers: MRE162 substr. FRA chromosome (CP119402), substr. FRA plasmid (CP119403), substr. NOR chromosome (P119400), substr. NOR plasmid (CP119401), substr. UK chromosome (CP119404), and substr. UK plasmid (CP119405). The associated BioSample accession numbers, including SRAs, are as follows: FRA (SAMN33417456), NOR (SAMN33417457), and UK (SAMN33417455), within BioProject PRJNA935550. The MRE162 UK clone has been deposited in the National Collection of Type Cultures (NCTC) under accession number 14920.
